# Effects of Gear Restriction on the Abundance of Juvenile Fishes along Sandy Beaches in Hawai‘i

**DOI:** 10.1371/journal.pone.0155221

**Published:** 2016-05-12

**Authors:** Mary K. Donovan, Alan M. Friedlander, Paolo Usseglio, Whitney Goodell, Ily Iglesias, Eva M. Schemmel, Kostantinos A. Stamoulis, Alexander Filous, Jonatha Giddens, Keith Kamikawa, Haruko Koike, Kaylyn McCoy, Christopher B. Wall

**Affiliations:** 1 Fisheries Ecology Research Lab, Department of Biology, University of Hawai‘i at Mānoa, Honolulu, HI, United States of America; 2 Pristine Seas, National Geographic Society, Washington, DC, United States of America; 3 Fundacion In-Nova, Castilla la Mancha, Toledo, Spain; 4 Department of Environment and Agriculture, Curtin University, Perth, Australia; 5 Hawai‘i Institute of Marine Biology, University of Hawai‘i at Mānoa, Honolulu, HI, United States of America; Department of Agriculture and Water Resources, AUSTRALIA

## Abstract

In 2007, due to growing concerns of declines in nearshore fisheries in Hawai‘i, a ban on gillnets was implemented in designated areas around the island of O‘ahu in the main Hawaiian Islands. Utilizing a 17 year time-series of juvenile fish abundance beginning prior to the implementation of the gillnet ban, we examined the effects of the ban on the abundance of juveniles of soft-bottom associated fish species. Using a Before-After-Control-Impact (BACI) sampling design, we compared the abundance of targeted fishery species in a bay where gillnet fishing was banned (Kailua, O‘ahu), and an adjacent bay where fishing is still permitted (Waimānalo, O‘ahu). Our results show that when multiple juvenile fish species were combined, abundance declined over time in both locations, but the pattern varied for each of the four species groups examined. Bonefishes were the only species group with a significant BACI effect, with higher abundance in Kailua in the period after the gillnet ban. This study addressed a need for scientific assessment of a fisheries regulation that is rarely possible due to lack of quality data before enactment of such restrictions. Thus, we developed a baseline status of juveniles of an important fishery species, and found effects of a fishery management regulation in Hawai‘i.

## Introduction

Fish populations around the world have declined dramatically in recent decades due to overfishing, land-based pollution, and habitat alteration [1–3]. This pattern is evident across all marine ecosystems, including nearshore tropical estuarine and soft-bottom habitats [4,5]. These nearshore ecosystems are especially vulnerable to anthropogenic impacts, as half of the world’s human population lives within 100 km of the shore [6]. Despite the close proximity of humans to these environments, nearshore soft-bottom habitats have not been studied as throughouly as other tropical ecosystems such as coral reefs. These habitats are particularily threatened by coastal development, land-use changes, and the effects of overfishing, and destructive fishing practices [5,7,8].

Specific gear types, such as gillnets, are of particular concern in nearshore soft-bottom habitats as they indiscriminately capture a wide range of species resulting in a large bycatch [9,10]. Bycatch can greatly outnumber targeted species and often contains undersized individuals, including juveniles of important target species, out-of-season species, and protected species [11]. Additionally, poor fishing practices such as leaving nets unattended for long periods of time, and lost nets that continue ‘ghost fishing’, contribute to the depletion of both targeted and non-targeted species [12]. Finally, the fishery can occur in remote locations and is often conducted at night, making enforcement of fishing regulations difficult and limiting the opportunity for fisheries-dependent sampling for research.

In Hawai‘i, nearshore fish populations have historically provided an important food resource, but over the past century there have been dramatic declines in the catch of important resource species, with the biomass of some species currently at levels < 1% of historical landings [13]. The use of gillnets is not found in the historical literature on Hawaiian cultural fishing practices, as they were introduced to Hawai‘i in the 1940-50s and subsequently proliferated across the state [14]. In Hawai‘i, gillnets are often referred to as ‘lay nets’ or ‘lay gill nets’, or by netting practices such as ‘set netting’, ‘cross netting’, ‘pa`ipa`i netting’, and ‘moemoe netting.’ These nets are inexpensive, commonly made of monofilament nylon with floats along one edge and weights along the opposite edge. In 2007, regulations were implemented to prohibit gillnets in certain areas of the island of O‘ahu due to the decline in the abundance and size of important resource species around the state and the perceived negative impact that gillnets have on these species and their habitats [15]. Thus, there is a need to better understand the status and trends of the fish communities associated with the gillnet fishery, and the effect of the gillnet regulations on the populations.

Juveniles of many important resource species utilize nearshore soft-bottom habitats [16]. Studies from other regions have shown that sandy beaches are important habitats for juvenile fishes, which provide foraging area and refuge from predators [17–22]. In Hawai‘i, utilization of inshore, estuarine, and soft-bottom habitats by juveniles of coastal resource species has been demonstrated for Pacific threadfin (*Polydactyls sexfilis*) [23,24], jacks (*Caranx melampygus* and *C*. *ignobilis*) [16] and mullet (*Mugil cephalus*) [25,26].

A number of important nearshore reef-associated fisheries species (e.g., mullet, goatfishes, jacks, bonefishes) are difficult to sample as adults using non-extractive methods due to their mobility, patchy distributions, and propensity for shallow habitats with low visibility [27]. However, many of these species aggregate in nearshore soft-bottom habitats as juveniles, thus providing an opportunity to assess the dynamics of their populations. Indices of juvenile abundance can act as early indicators of future adult stock recruitment and document annual variation and long-term trends in abundance and distribution [28,29]. For this study, we assumed that if the gillnet ban had a positive effect on the abundance and size of a given species, this should have resulted in greater reproductive output and thus increases in the number of juveniles and adults over time [30]. Additionally, banning gillnets could directly increase juvenile survival through reduced fishing mortality, as studies have shown that juveniles can comprise nearly half of the catch in some gillnet fisheries [31–33].

**Fig 1:**
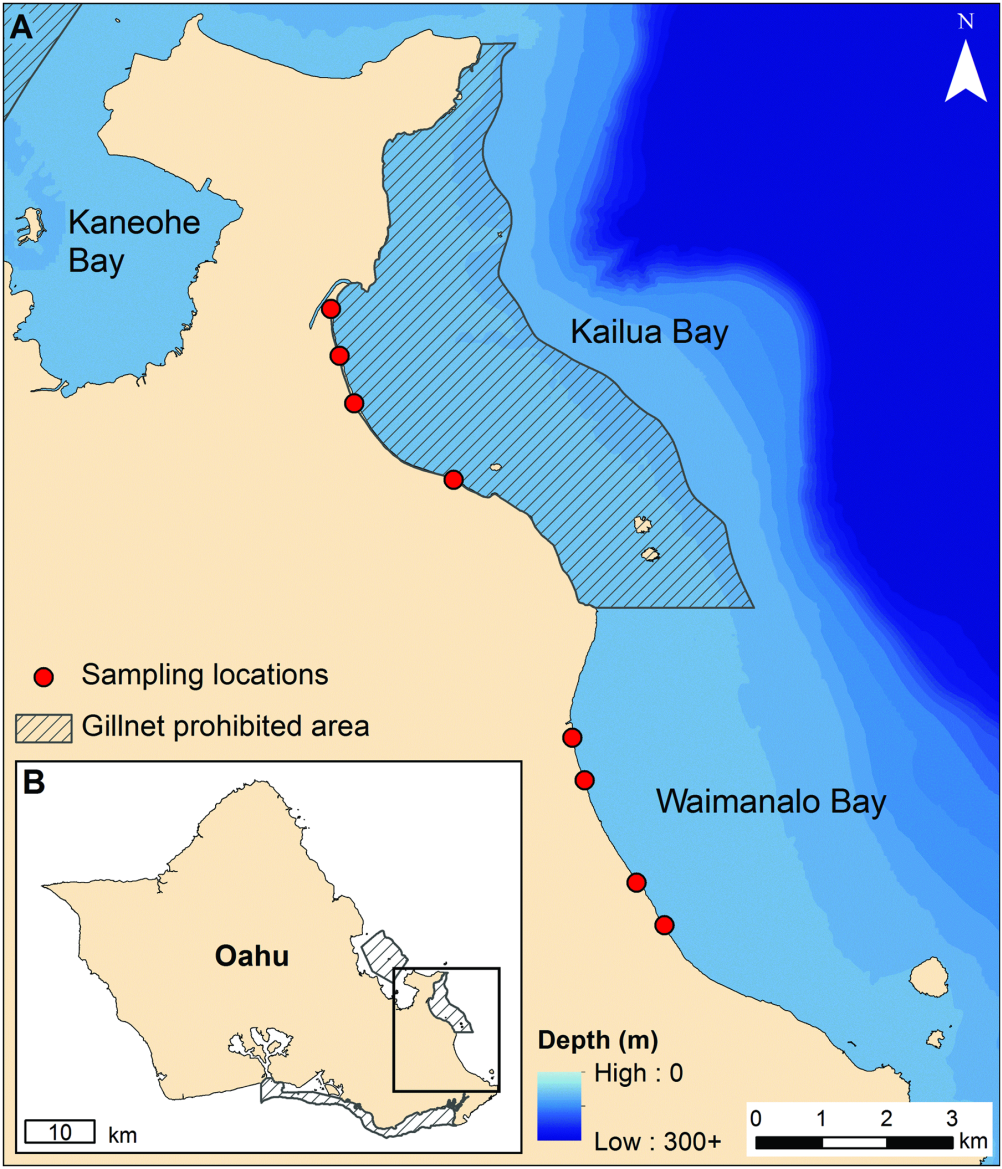
Map of study area showing gillnet prohibited area (hatched area) in Kailua Bay and open area in Waimanalo Bay (A) on the island of O‘ahu in Hawai‘i (B). Red circles show sampling locations, and depth contours are colored from light (shallow) to dark (deep).

In order to measure the effect of any fisheries management strategy, it is extremely valuable, but uncommon, to have long-term data from before the regulations were enacted. This study is unique in that we analyse a 17-year dataset on the abundance of fishes in the surf zone along sandy beaches on windward O‘ahu starting in 1997, 10 years prior to the implementation of a management action to restrict the use of gillnets [24]. Due to the existence of these data prior to the implementation of the gillnet ban, we were able to examine the effects on the abundance of soft-bottom fish species in a bay where gillnet fishing has been banned (Kailua), and a similar nearby bay where gillnet fishing is still permitted (Waimānalo) (Fig 1). We utilized a Before-After-Control-Impact (BACI) sampling design, which is a powerful statistical approach that considers the natural differences between control and impact locations and the changes due to an event (impact) that influences both sites the same way [34,35]. The objective of this study was to assess differences in juvenile fish populations between a protected and an unprotected area, before and after the gillnet closure, to quantify and contrast changes in juvenile fish abundance due to these regulations.

## Materials and Methods

### Ethics statement

This work was permitted by the Hawai‘i Department of Land and Natural Resources Division of Aquatic Resources under Special Activity Permit No. 2010–24 to the Oceanic Institute and No. 2012–70 to the University of Hawai‘i.

### Surveys

On the island of O‘ahu, gillnet fishing has been prohibited in Kailua Bay since 2007 (Fig 1). Kailua and the adjacent bay of Waimānalo, where gillnet fishing is allowed, represent an ideal paired sample design since these locations have similar physical and environmental characteristics. The two bays have the same direction of exposure to wind and waves, however the offshore bathymetry is steeper in Waimānalo, allowing for greater attenuation of wind waves from NE trade winds compared to Kailua (Fig 1). Although it is difficult to control for other methods of fishing, the impact of spear, pole-and-line, and other types of fishing typical of the area is likely modest compared to the impact of gillnets, which have catch rates an order of magnitude greater than most other gear types used in Hawai‘i [36–38].

At each site, four replicate substations were surveyed monthly from January 1997 to December 2013 (Kailua: -157.7445, 21.4218; -157.7433, 21.4154; -157.7412, 21.4088; -157.7266, 21.3981, Waimanalo: -157.6961, 21.3364; -157.7001, 21.3423; -157.7077, 21.3565; -157.7094, 21.3624). At each substation, three beach seine hauls were conducted using a 24 x 1.8-m long seine net, with a square mesh size of 0.5 cm. Each haul was conducted in the surf zone with one person positioned at each end of the net and a third person tending the net’s cod-end, ensuring the cod remained open and did not rise off the bottom allowing fish to escape. Hauls were conducted perpendicular to shore, starting with the seine stretched fully open at a standardized depth of 1.5 meters (average sample area = 360 m^2^). The mean haul length was 5 minutes, and varied by a standard deviation of 2 minutes depending on current and sea state. Processing consisted of placing fishes in a tub of seawater identifying each individual to species or species groups, recording fork length, and total number of each species. All fishes were released back to the water after all three replicate hauls were complete at each substation.

### Analysis

Four species groups were evaluated in the analysis, as they were consistently present in the samples and are known to be important to the gillnet fishery in Hawai‘i. The species included in our analyses were bonefishes (‘o‘īo; *Albula virgata and A*. *glossodonta*), Pacific threadfin (moi), flagtails (āholehole; *Kuhlia xenura and K*. *sandvicensis*), and jacks (pāpio; *Caranx* spp.). Other commonly sampled taxa, important as food fishes, were mullet (Mugilidae) and goatfishes (‘oama; Mullidae), but their abundance and frequency of occurrence were not sufficient to allow for accurate modeling of abundance and were therefore excluded from further analysis. As the focus of this study was on juveniles, counts of individuals > 300 mm for bonefishes, > 150 mm for Pacific threadfin, > 120 mm for flagtails, and > 200 mm for jacks were discounted in the analysis based on published size estimates [23,39–41]. Species were combined into species groups due to inaccuracies in identifying species at small sizes, and differences in taxonomic resolution of observations throughout the time series.

**Fig 2:**
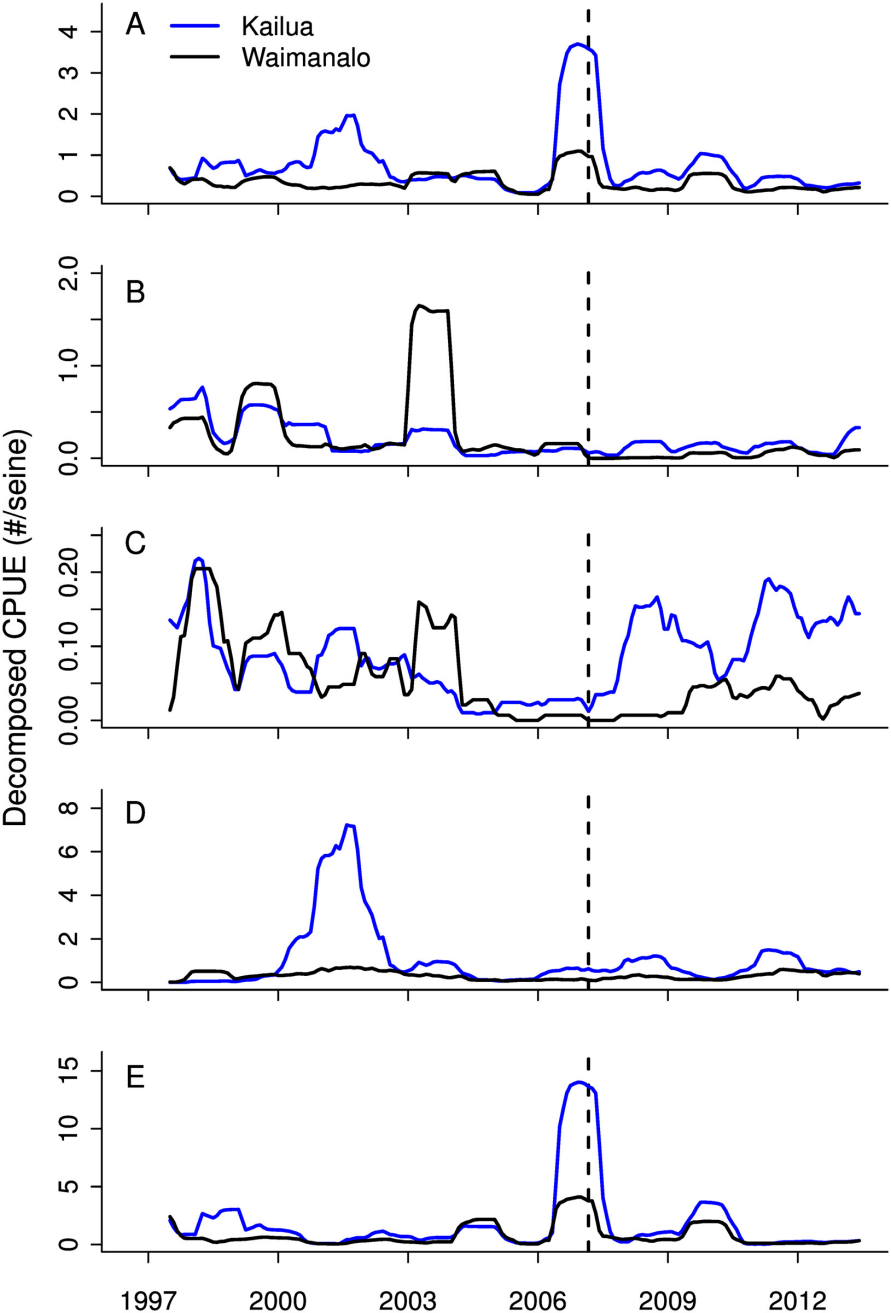
Trend component of decomposed time series of catch per unit effort (CPUE) at two locations, Kailua (blue) and Waimānalo (black), for (A) all species combined, (B) jacks, (C) bonefishes, (D) flagtails, and (E) Pacific threadfin. Vertical line represents gillnet ban in Kailua in 2007

Mean catch rate by site was calculated as the mean of the total number of individuals divided by the total number of hauls for each substation (hereafter catch-per-unit-effort [CPUE]). The abundance of juvenile fishes can be highly variable and dependent on seasonality associated with the timing of spawning and reproduction, as well as local environmental conditions. Given this, we constructed a time-series model using the ‘decompose’ function in the *stats* package in R [42], allowing us to differentiate three components of the time-series including the trend, a seasonal signal, and a random component, using a moving window with an additive model. The trend component was then extracted and used as the response variable in subsequent analyses. The trend component was plotted against time for each species group individually and as an aggregate of all four species groups combined (Fig 2).

General linear mixed effects models were used to test changes in the trend component of decomposed mean abundance between the two locations for all species combined and for individual species using a Before-After-Control-Impact (BACI) design where the data were classified as ‘before’ and ‘after’ the effective date of the fishery closure on March 2, 2007 [15,43,44]. The response variable (decomposed trend of CPUE) was modeled with a Gaussian distribution [45]. We accounted for spatial and temporal dependence by adding random components of period (before/after) nested within each site (Kailua/ Waimānalo), thus each replicate (mean by site and month) was treated as a repeated measure and the full model form was decomposed CPUE ~ site + period + site*period + (1|site/period/Julian month). Model fits were assessed by visual inspection of the residuals, and a maximum likelihood estimate was used to fit the models. Mixed models were constructed with packages *lme4* [46] and *glmmADMB* [47,48], using the software program R version 3.1.1 [42]. Likelihood ratio tests were used for hypothesis testing with the ‘Anova’ function in the package *car* [49].

Differences in size distributions were compared across sites for juveniles of each species using species-specific maximum size classes, defined above. Size frequency histograms were created with 20 mm bins, and comparisons of mean size by species were tested with individual linear models.

## Results

Between January 1997 and December 2013, 5878 beach seine hauls were conducted in Kailua and Waimānalo Bays. A total of 49 taxa were sampled overall, but four species groups represented 88% of all fishes sampled. Flagtails were the most frequently encountered species group, occurring in 34% of the samples, followed by Pacific threadfin (30%), jacks (13%), and bonefishes (11%).

Trends in abundance were variable over the study period, with evidence of several large recruitment events marked by peaks in abundance (at least one standard deviation above the mean; Fig 2). The abundance of jacks showed a peak in 1999 in both Kailua and Waimānalo and again in 2003 in Waimānalo, with persistently low abundance for the remainder of the study period (Fig 2B). The abundance of bonefishes was the most variable of the four species groups, with frequent peaks in both locations (Fig 2C), and a notable rise in abundance in Kailua after 2007. Flagtail abundance was generally consistent across the time series, with the exception of a large peak from November 2000 –March 2002 in Kailua (Fig 2D). Pacific threadfin abundance was also generally consistent, with one large peak in Kailua Bay from June 2006 –June 2007 (Fig 2E). This peak was mirrored in Waimānalo but with a smaller magnitude.

**Fig 3:**
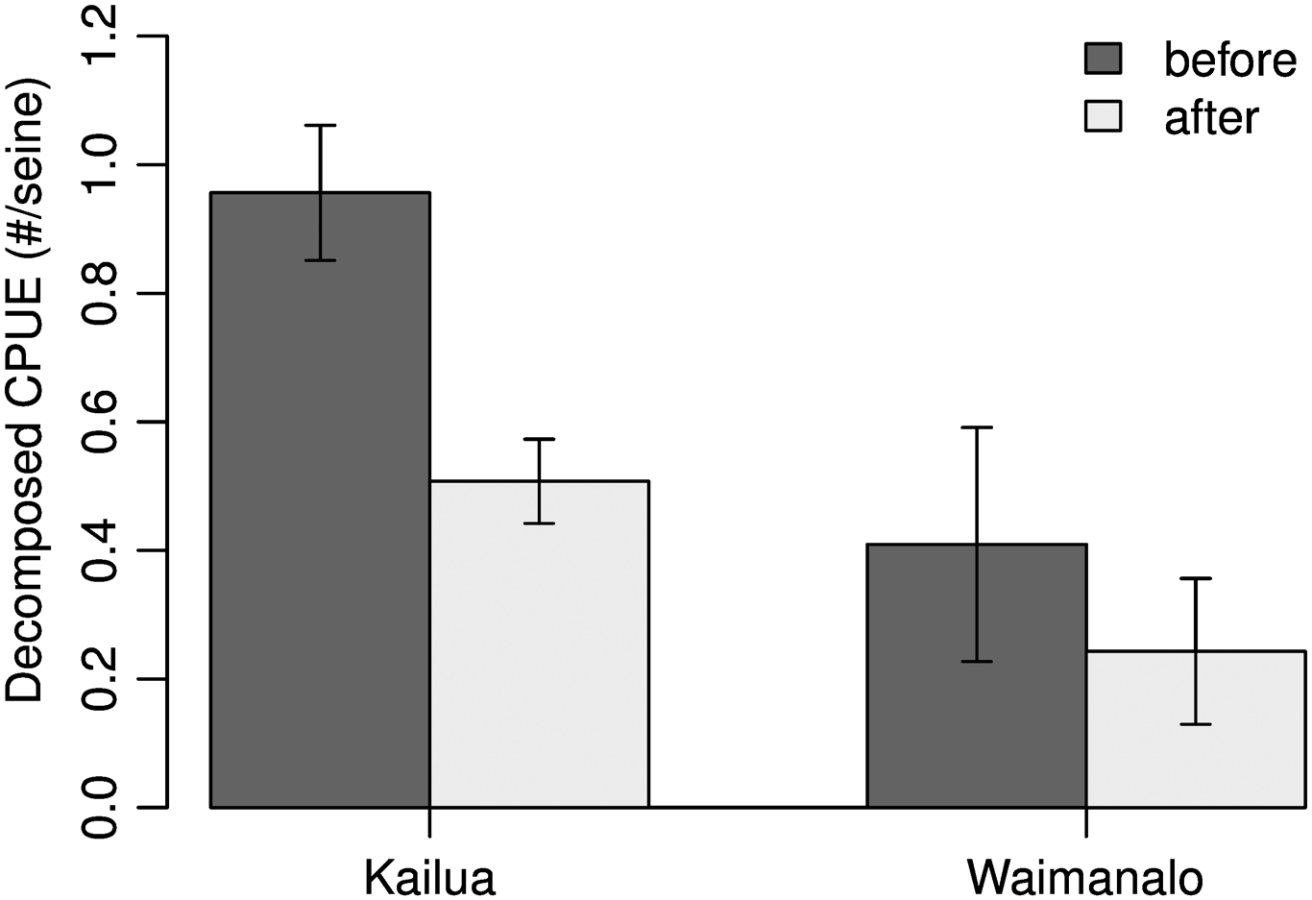
Results of general linear mixed models of catch per unit effort (CPUE) for all species combined following a Before-After-Control-Impact (BACI) design to measure differences in juvenile fish populations between a protected area (Kailua) and an unprotected area (Waimānalo), before and after a gillnet closure in Hawai‘i (error bars ± 1 SE).

When all species were combined into a single analysis, no BACI effect was found (Table 1: All fishes Site*Period = -0.28, $X_{1,377\;}^{2}$ = 5.91, *p* = 0.02; Figs 2A and 3), however significant declines in abundance were observed in both bays following the closure ($X_{1,377\;}^{2}$ = 29.8, *p* < 0.01). If a BACI effect was present the Site*Period coefficient would be positive, and the Site coefficient would be negative [43]. However large differences in species-specific patterns were evident (Fig 2), and combining species into a single analysis obscured patterns related to the relative abundance of different species groups.

**Table 1:** Results of general linear mixed models of catch per unit effort (CPUE) for all species combined and for individual species following a Before-After-Control-Impact (BACI) design to measure differences in juvenile fish populations between a protected and an unprotected area, before and after a gillnet closure in Hawai‘i.

	Predictor	Coef *B*	SE(*B*)	*z*	*X*^*2*^	*p*
All fishes						
	Intercept	0.51	0.07	7.75		<0.01
	Site	-0.27	0.09	-2.86	8.15	0.01
	Period	0.45	0.08	5.46	29.80	<0.01
	Site*Period	-0.28	0.12	-2.43	5.91	0.02
Jacks						
	Intercept	0.13	0.03	3.99		<0.01
	Site	-0.09	0.05	-1.95	3.79	0.05
	Period	0.11	0.04	2.62	6.85	<0.01
	Site*Period	0.20	0.06	3.42	11.72	<0.01
Bonefishes						
	Intercept	0.12	0.01	22.66		<0.01
	Site	-0.10	0.01	-12.40	153.97	<0.01
	Period	-0.06	0.01	-8.62	74.29	<0.01
	Site*Period	0.10	0.01	10.23	104.59	<0.01
Flagtails						
	Intercept	0.74	0.13	5.77		<0.01
	Site	-0.43	0.18	-2.36	5.58	<0.01
	Period	0.49	0.16	3.05	9.28	0.01
	Site*Period	-0.49	0.23	-2.15	4.63	0.03
Pacific threadfin						
	Intercept	0.94	0.26	3.57		<0.01
	Site	-0.38	0.37	-1.03	1.05	0.30
	Period	1.31	0.33	3.96	15.65	<0.01
	Site*Period	-0.98	0.47	-2.09	4.40	0.04

**Fig 4:**
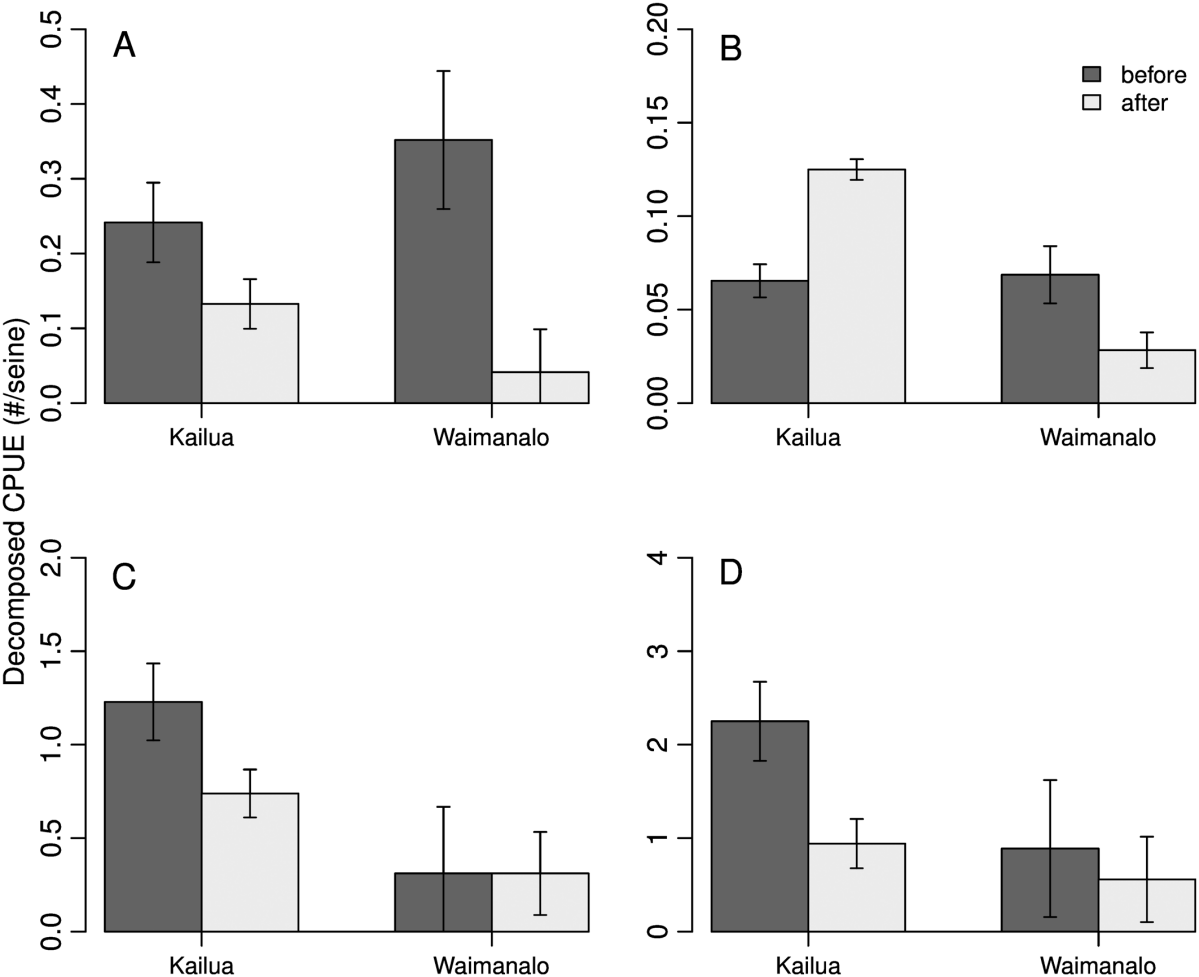
Results of general linear mixed models of catch per unit effort (CPUE) for (A) jacks, (B) bonefishes, (C) flagtails, and (D) Pacific threadfin following a Before-After-Control-Impact (BACI) design to measure differences in juvenile fish populations between a protected area (Kailua) and an unprotected area (Waimānalo), before and after a gillnet closure in Hawai‘i (error bars ± 1 SE).

When bonefishes were considered independently, a significant BACI effect was found (Table 1, Figs 2 and 4). There was a significant interaction between site and period (Fig 4B; $X_{1,377\;}^{2}$ = 104.59, *p* < 0.01), and a significantly greater abundance in Kailua but not Waimānalo following the closure ($X_{1,377\;}^{2}$ = 153.97, *p* < 0.01).

Results for the other species groups were less consistent. The abundance of jacks declined in both bays following the fishery closure (Fig 4A; $X_{1,377\;}^{2}$ = 3.79, *p* = 0.05), with the only large peaks in abundance occurring early in the study period (Fig 2B). The abundance of flagtails was significantly greater in Kailua than in Waimānalo in both periods (Fig 4C; $X_{1,377\;}^{2}$ = 4.63, *p* = 0.03), and within Kailua declined following the closure ($X_{1,377\;}^{2}$ = 9.28, *p* = 0.01). Pacific threadfin abundance declined in Kailua after the fishery closure ($X_{1,377\;}^{2}$ = 15.65, *p* < 0.01), and was not significantly different in Waimānalo across the two periods (Fig 4D; $X_{1,377\;}^{2}$ = 1.05, *p* = 0.30).

**Fig 5:**
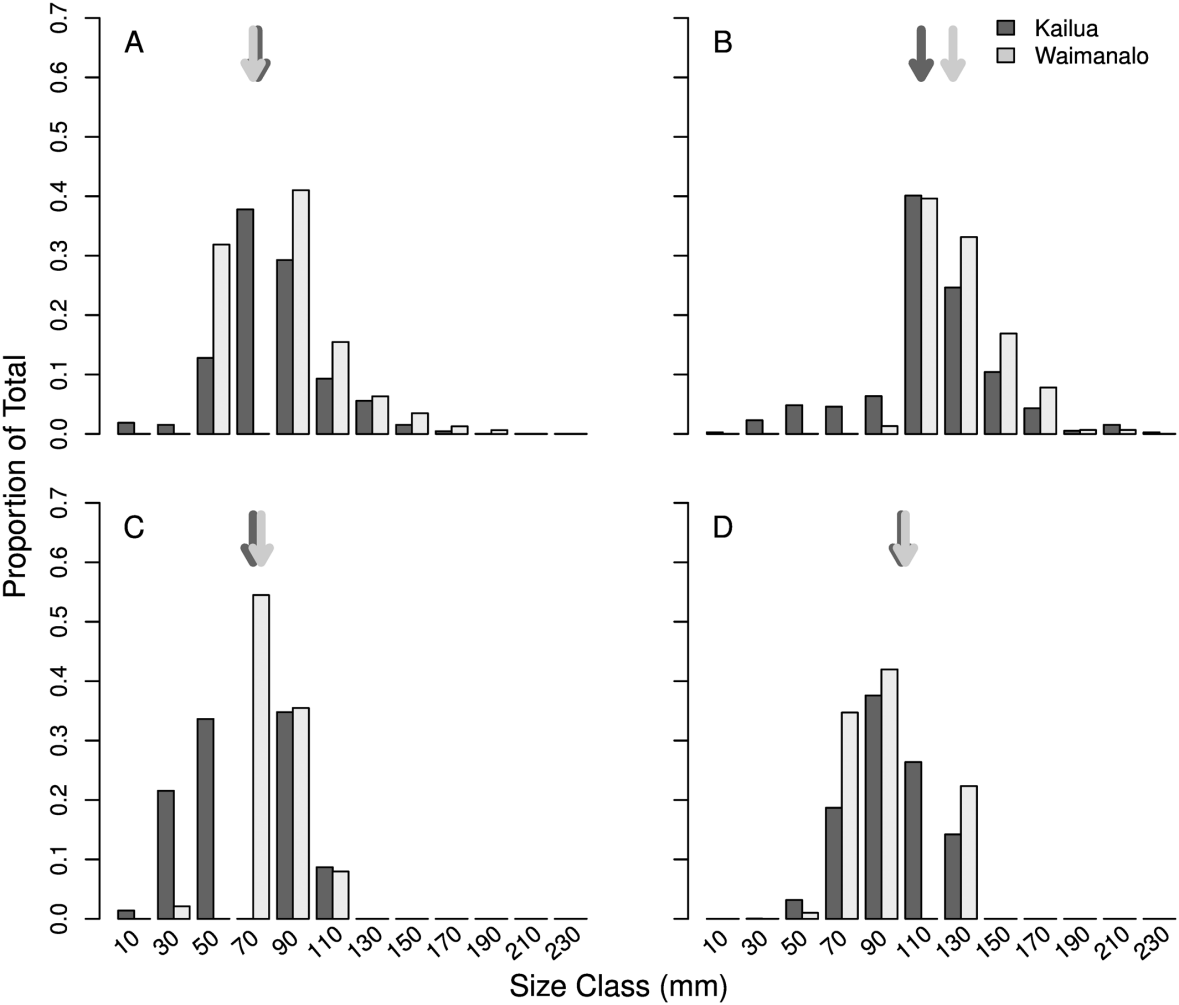
Length frequency distribution for (A) jacks, (B) bonefishes, (C) flagtails, and (D) Pacific threadfin between a protected area (Kailua—light grey) and an unprotected area (Waimānalo—dark grey). Vertical arrows correspond to mean length at each site.

Sizes of juvenile jacks ranged from 17 to 180 mm in Kailua, and 51 to 194 mm in Waimānalo and there was no significant difference in the mean size across sites (Fig 5A; Kailua = 89.4 mm, Waimānalo = 88.0, F_1,1515_ = 1.22, *p* = 0.27). Sizes of juvenile bonefishes ranged from 27 to 239 mm in Kailua, and 105 to 221 mm in Waimānalo, with a significantly greater mean size in Waimānalo (Fig 5B; Kailua = 124.1 mm, Waimānalo = 137.9, F_1,546_ = 24.6, *p* < 0.01). Sizes of juvenile flagtails ranged from 15 to 120 mm in Kailua, and 40 to 120 mm in Waimānalo, with a significantly greater mean size in Waimānalo (Fig 5C; Kailua = 74.9 mm, Waimānalo = 80.9, F_1,4053_ = 60.94, *p* < 0.01). Sizes of juvenile Pacific threadfin ranged from 48 to 149 mm in Kailua, and 54 to 149 mm in Waimānalo and there was no significant difference in the mean across sites (Fig 5D; Kailua = 105.2 mm, Waimānalo = 106.2, F_1,6901_ = 3.6, *p* = 0.06).

## Discussion

Our results show that the abundance of juvenile bonefishes was higher in Kailua Bay, where gillnets were banned in 2007, compared to nearby Waimānalo Bay, where gillnet fishing is still allowed after the closure. Many of the species targeted in the gillnet fishery are highly mobile, schooling fishes, and some exhibit considerable seasonal and inter-annual variability [23,41,50,51]. These characteristics make it inherently difficult to estimate trends in abundance. However, the approach taken in this study overcomes many of these difficulties by utilizing time series analysis in conjunction with hierarchical statistical models to account for variability due to seasonality and sampling effects. While there are several limitations to extrapolating results to other areas, this study is an important demonstration of increased abundance of a target species following a fisheries management regulation. In addition, this study is unique in that the duration of the 17-year dataset used is unprecedented for juvenile nearshore fishes in a soft-bottom habitat.

**Fig 6:**
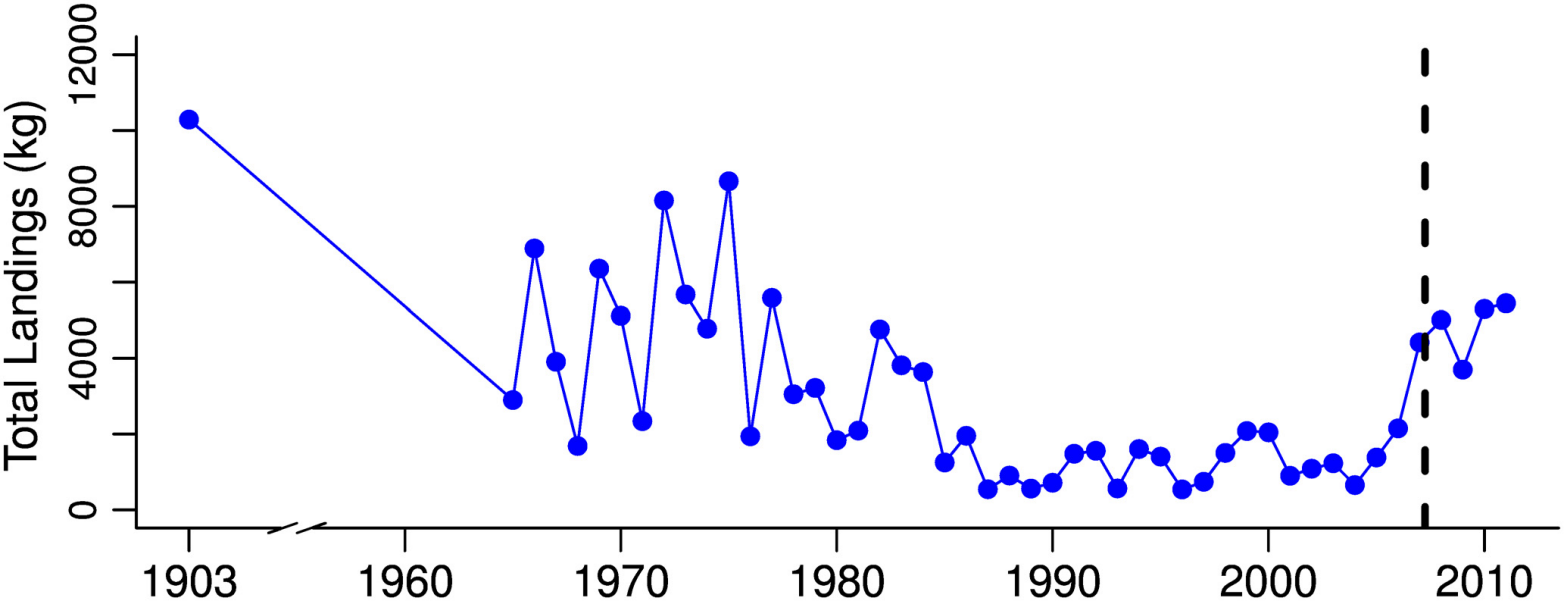
Commercial landings of bonefishes from 1900, and 1965–2011 for the island of O‘ahu. Vertical line represents gillnet ban in Kailua in 2007. Data from 1903 from [55], and otherwise from Hawai‘i Division of Aquatic Resources.

Increases in juvenile abundance of bonefishes following six years of protection from gillnetting may have resulted from several non-mutually exclusive processes, including: decreased adult mortality leading to increased stock size and greater reproductive output and recruitment, and decreased juvenile mortality [52]. For the system examined here, an increase in spawning stock as a direct result of the gillnet ban would assume that the juveniles observed in the study area were the result of self-recruitment or larval export from other gillnet protected areas such as Kāne‘ohe Bay or O‘ahu’s south shore (Fig 1). For bonefishes, this may be unlikely given their long pelagic larval duration (up to 72 days [53]). However, evidence of spatially dependent patterns of recruitment of bonefishes was found in the Bahamas at a scale of 5 km [54], which is commensurate with the scale of this study (Fig 1). Also, the increase in abundance of juvenile bonefishes in Kailua Bay observed in this study corresponds with an increase in the total commercial landings of bonefishes on the island of O‘ahu over the same period (Fig 6). While it may not be appropriate to directly ascribe island-level increases in landings to a single gillnet restriction area, the observed increases in Kailua Bay may be indicative of the important role played by gillnet restricted areas around the island in rebuilding bonefish stocks.

The bonefish fishery is distinct compared to that of other species examined in this study. Historically, bonefishes played an important cultural role in Hawai‘i [56] and represented a significant proportion of the commercial catch [13]. Today, both the commercial fishery and recreational fisheries are growing [51], however species-specific management for bonefishes is limited. A minimum size of 36 cm was established in 2010 [57], but this rule does not distinguish between the two bonefish species found in Hawai‘i (the smaller of which is endemic to Hawai‘i) and the size at maturity for both species exceeds the regulated minimum size [39]. At the same time as the gillnet restricted areas were established in 2007, net regulations were also implemented, setting a minimum mesh size of 2.75 in (7.00 cm), and a maximum net size of 125 feet (38.10 m) long and 7 feet (2.13 m) high [15]. Therefore, it is possible that the trends observed in this study could be related to decreased mortality of juvenile fishes after these rules were implemented. However, if this were the sole mechanism and the gillnet restricted area did not play a role, we would expect to have observed an effect at both sites since the additional net regulations applied to both areas.

The life history of the bonefish species could also have contributed to the observed patterns in this study. Bonefishes in Hawai‘i have high site fidelity, and are commonly recaptured within one kilometer of a prior catch location [51], which could explain why an area such as Kailua Bay might serve as an important refuge for this species. Importantly, the two bonefish species in Hawai‘i have different habitat preferences, and different vital rates [39]. While the two species were not differentiated in the analyses due to inconsistent identification along the duration of the study, where it was possible to compare species, over 83% of observed bonefish were the endemic species (*A*. *virgata*). Compared to *A*. *glossodonta*, *A*. *virgata* inhabit soft bottom habitats as adults more frequently, so it is possible that this difference in habitat preference is similar in the juvenile stage as well. Mean size across sites was significantly different for bonefishes (Fig 5), with the smaller size classes (< 90 mm) only observed in Kailua, which could relate to differences in habitat between bays.

Finally, it is possible that changes in human behavior and fishery usage affected fish abundance at Kailua and Waimānalo Bays. Restricting gillnetting in certain areas may simply displace fishing activities to nearby sites without restrictions, or lead fishers to adopt other capture methods, so further study could assess changes in fisheries activities following the gillnet ban. Given the large variation in patterns among the four species groups examined, this study highlights the need for considering species-specific effects of management regulations. Overall declines in juvenile fish abundance for all other species besides bonefishes during our 17 year survey period indicate that fishing intensity may exceed the ability of local fish populations to replenish despite regulating gillnets in certain areas.

Juvenile fish recruitment exhibits high spatial and temporal variation, and it can be difficult to say with certainty that changes in abundance are attributable to changes in fisheries management or other intrinsic factors (i.e., ocean currents, population connectivity, recruitment substrata). The two bays examined in this study have comparable exposure to wind and waves. However, differences in bathymetry allow larger waves to reach shore in Waimānalo Bay, which could have resulted in a bias in our sampling due to the limitations of the beach seine method in high impact surf zones. Likewise, this study did not employ a formal CPUE standardization to control for latent effects that could bias the use of catch as a proxy for abundance [58]. Time series analysis was used to isolate the trend portion of the data, and dampen patterns due to stochasticity and seasonality in the observations. Additionally, with a BACI design we were able to separate the effects of site and time and investigate how changes in a management action resulted in changes in abundance of juvenile fishes. We further accounted for variation in location by nesting our sampling design in a mixed-effects model and the resulting variation due to the random effect of site was orders of magnitude smaller than the variation due to the BACI effect (Table 1). It is possible that these techniques were not adequate to detect a signal for all species groups, but we were able to detect regulation-associated patterns for at least one important resource species group.

Research on the life histories, habitat associations, and correlations with physical forcings such as rain, wind, waves, and tides would also be valuable to help explain variability in these dynamic nearshore fish populations. For example, many of the species consistently caught during our study are known to rely on freshwater habitats during their early life stages, and future research could inform our understanding of these associations and their impact on survival to adulthood. We have discussed several limitations to the study, however our results show a clear trend for increasing abundance of at least one species group of fishes (bonefishes) at a site where gillnetting was restricted relative to a site where it still occurred, providing compelling evidence for the effectiveness of the gear restrictions in rebuilding an important fishery in Hawai‘i.
